# Patterns of Expansion and Expression Divergence of the Polygalacturonase Gene Family in *Brassica oleracea*

**DOI:** 10.3390/ijms21165706

**Published:** 2020-08-09

**Authors:** Meiling Lyu, Junaid Iftikhar, Rongfang Guo, Binghua Wu, Jiashu Cao

**Affiliations:** 1 College of Horticulture, Fujian Agriculture & Forestry University, Fuzhou 350002, China; mllyu@fafu.edu.cn (M.L.); junaidiftikhar@outlook.com (J.I.); 000q813038@fafu.edu.cn (R.G.); binghua.wu@fafu.edu.cn (B.W.); 2 Laboratory of Cell & Molecular Biology, Institute of Vegetable Science, Zhejiang University, Hangzhou 310058, China; 3 Zhejiang Provincial Key Laboratory of Horticultural Plant Integrative Biology, Zhejiang University, Hangzhou 310058, China

**Keywords:** *Brassica oleracea*, polygalacturonase, gene family, evolution, expression analysis

## Abstract

Plant polygalacturonases (PGs) are closely related to cell-separation events during plant growth and development by degrading pectin. Identifying and investigating their diversification of evolution and expression could shed light on research on their function. We conducted sequence, molecular evolution, and gene expression analyses of PG genes in *Brassica oleracea*. Ninety-nine *B. oleracea* PGs (BoPGs) were identified and divided into seven clades through phylogenetic analysis. The exon/intron structures and motifs were conserved within, but divergent between, clades. The second conserved domain (GDDC) may be more closely related to the identification of PGs. There were at least 79 common ancestor PGs between *Arabidopsis thaliana* and *B. oleracea*. The event of whole genome triplication and tandem duplication played important roles in the rapid expansion of the BoPG gene family, and gene loss may be an important mechanism in the generation of the diversity of BoPGs. By evaluating the expression in five tissues, we found that most of the expressed BoPGs in clades A, B, and E showed ubiquitous expression characteristics, and the expressed BoPGs in clades C, D, and F were mainly responsible for reproduction development. Most of the paralogous gene pairs (76.2%) exhibited divergent expression patterns, indicating that they may have experienced neofunctionalization or subfunctionalization. The cis-elements analysis showed that up to 96 BoPGs contained the hormone response elements in their promoters. In conclusion, our comparative analysis may provide a valuable data foundation for the further functional analysis of BoPGs during the development of *B. oleracea*.

## 1. Introduction

Pectin is the main component of the primary cell wall. Its degradation happens in a variety of periods of plant development, such as seed germination, organ abscission, fruit ripening, anther dehiscence, silique dehiscence, pollen development, pollen tube growth, and so on [1,2,3,4]. Polygalacturonases (PGs) are cell wall hydrolases. They can catalyze random hydrolysis and disassembly of the 1,4-α-D-galactosiduronic linkages of pectin, causing pectin degradation [5]. Therefore, PGs participate in almost all of the developmental processes of plants. In *Arabidopsis thaliana*, more than 50% of PGs with high expression levels were found in floral tissues, suggesting their important roles in flower development [6]. In the process of the maturation and shedding of oil palm fruit, 14 endogenous PG genes were identified by the transcriptional analysis, and these genes were characterized in the shedding area when the fruit was ripe [7]. Genome-wide identification and analysis of PGs in *Solanum lycopersicum* showed that most SlPGs had specific or high expression patterns in at least one organ, and five PGs were associated with fruit development [8]. In *Brassica rapa*, the PGs BcMF2, BcMF6, BcMF16, BcMF17, and BcMF24 have been shown to play important roles in pollen development and pollen tube growth [9,10,11,12,13]. The PG gene *BoMF25* in *Brassica oleracea* was proven to be related to the development of the pollen wall [14]. Recently, He et al. [15] characterized the maize PG gene *ZmPGH1* and demonstrated that it functions as a suppressor of programmed cell death in plants.

PGs belong to a large gene family in plants. In *A. thaliana*, *Cucumis sativus*, *Citrullus lanatus*, *Populus*, *Oryza sativa*, *B. rapa*, and *Malus sieversii*, the PG gene family members have been widely identified and analyzed based on their genomes [6,16,17,18,19,20,21,22]. According to clustering maps or expression characteristics, PGs are divided into several clades (A-C or A-F). Studies have shown that different PG members in the same clades have high similarity in terms of their gene structure and molecular characteristics [18]. A whole genome duplication (WGD) event occurs when accompanied by the fractionation and rearrangement of genome fragments, providing initial resources for genome evolution and gene differentiation, and resulting in the gain and loss of PG genes. WGD and tandem duplication events also generated many duplicated PG genes [23,24]. During the evolutionary process, most PG duplicates have partially overlapping but distinct expression patterns in different tissues and organs [6]. The differences in expression regulation patterns between different members are mainly determined by three regulatory factors: the promoter sequence, cis-regulatory element, and exon-intron structure [25,26,27]. An in-depth analysis of the composition, expansion, and expression patterns of the PG gene family has far-reaching significance for its evolution and function.

During the evolution process, Brassicaceae species underwent two WGD events and one Brassicaceae lineage-specific whole-genome triplication (WGT) event, which involved substantial genome reshuffling and gene loss [28,29,30]. Therefore, Brassicaceae species comprise a good model for the study of polyploid genome evolutionary mechanisms. In the current study, we conducted genome-wide identification and analysis of PG genes in the *B. oleracea* genome. Through a comprehensive analysis of the gene sequences, gene structures, molecular evolution, a single gene, and duplicated gene expression patterns, as well as subcellular localization of PG proteins, we provided a detailed characterization of the composition, expansion, and expression divergence of the PG gene family in *B. oleracea*. The results will offer valuable data for studying the evolution and biological functions of PGs in plants.

## 2. Results

### 2.1. The Polygalacturonase Gene Family in B. oleracea Consists of 99 Members

Ninety-nine full-length genes encoding putative PG proteins were identified in the *B. oleracea* (Supplementary Materials Table S1). Using Pfam, the candidates were confirmed as having GH28 domains in the protein structure.

According to the sequence characteristics analysis (Supplementary Materials Table S2), the open reading frames (ORF) of *B. oleracea* PGs (BoPGs) were from 555 bp (*Bol030036*) to 1650 bp (*Bol007591*) in length. The length range of the amino acid sequence was 184 to 549 aa, and the corresponding molecular weight ranged from 19.7 to 59.1 kDa. Their isoelectric point range was 4.83 (Bol011049) to 8.52 (Bol021650). The signal peptide prediction results of the amino acid sequence showed that they all contained an N-terminal signal peptide with a length from 10 (Bol044586) to 58 aa (Bol013164), which indicated that they were all secreted proteins.

**Figure 1:**
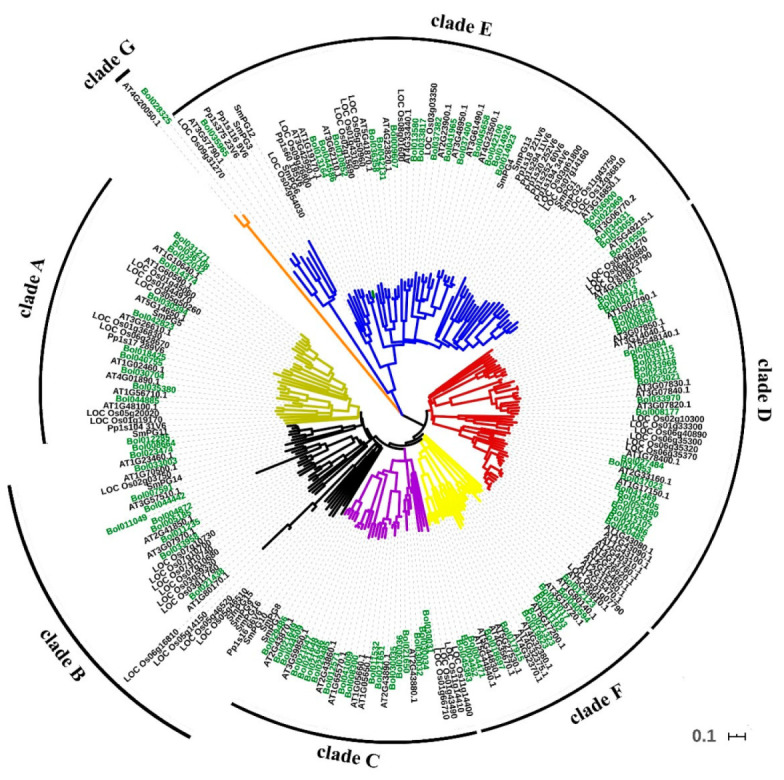
Phylogenetic tree of the 238 polygalacturonases (PGs) from five land plant species. The tree was constructed by using the neighbor-joining (NJ) method. The bar indicates the relative divergence of the sequences examined.

To investigate the relationships between PGs, we performed a phylogenetic analysis of five land plant species, namely *A. thaliana*, *B. oleracea*, *O. sativa, Selaginella moellendorffii* (spikemoss, lycophyte), and *Physcomitrella patens* (moss, bryophyte). All 238 PGs fell into seven distinct clades (A–G) (Figure 1). We counted the PGs of different clades in these five species (Supplementary Materials Table S3). The results showed that the PGs of dicotyledonous plants *A. thaliana* and *B. oleracea* were distributed in A~G clades, and the clade G only contained PG genes of dicotyledonous plants. The PGs of monocotyledonous rice were distributed in A~F clades. According to our result, the number of members of the PG gene family increased rapidly from monocotyledons to dicotyledons. Compared with the number in the *O. sativa* genome, the PG members increased by 24 in *A. thaliana* and by up to 55 in *B. oleracea*. Meanwhile, a marked difference was also found in the PG gene family size among bryophyte, lycophyte, and angiosperms. The *P. patens* and *S. moellendorffii* PGs only existed in clades A, B, and E.

**Figure 2:**
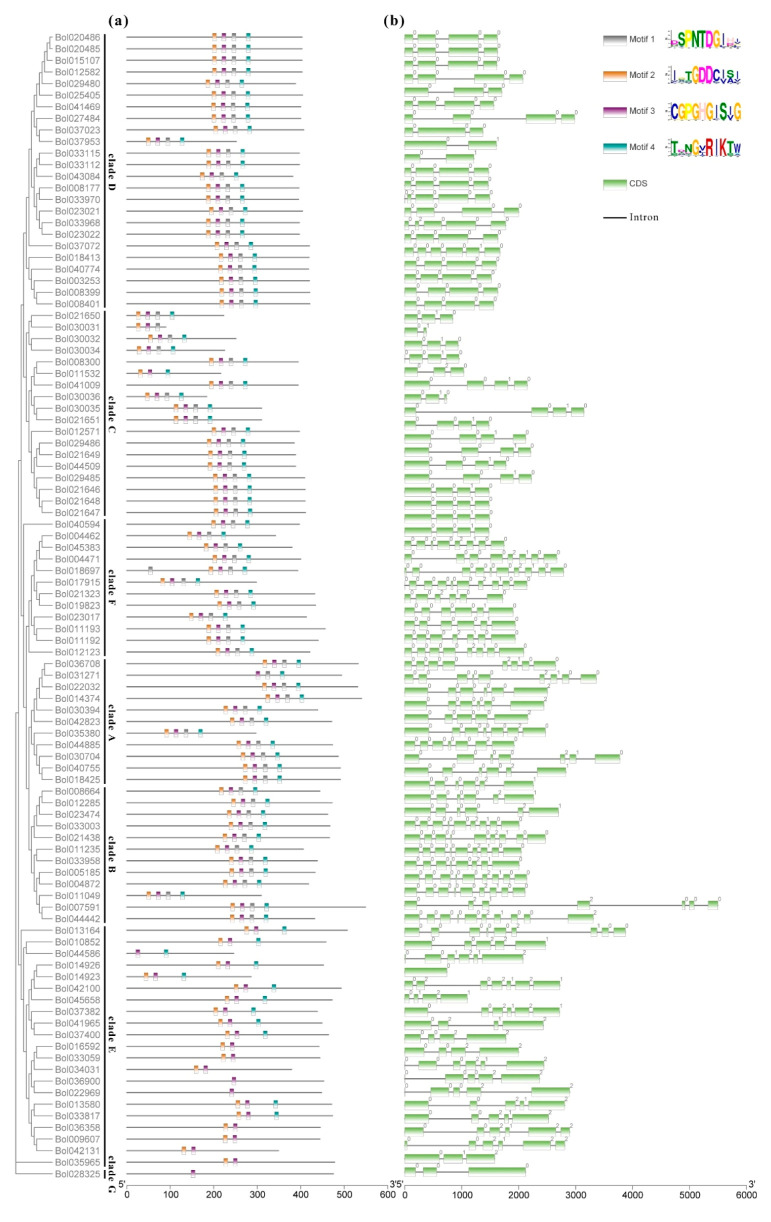
Analysis of the conserved motifs and gene structures of *Brassica oleracea* PGs (BoPGs). (**a**) Four motifs of PG genes were investigated by the online tool MEME. Motif I to motif IV (I, SPNTDGI; II, GDDC; III, CGPGHGIS; and IV, RIK) are indicated by different color boxes, respectively. (**b**) Exon and intron organization of PG genes. Exons and introns are represented by green boxes and black lines, respectively. Intron phases are also analyzed from the exon information. Phase 0 is designated as introns between codons, phase 1 is designated as introns between the first and the second nucleotide in a codon, and phase 2 is designated as introns between the second and third nucleotides in a codon.

One gene containing at least one of the four conserved domains (I, SPNTDGI; II, GDDC; III, CGPGHGIS; and IV, RIK) can be identified as PG. The conserved domain analysis revealed that almost all of the BoPGs in clades A, B, C, D, and F contained all of the four conserved domains of BoPG (Supplementary Materials Table S2 and Figure 2a). The II domain was the most conserved domain, and it could be found in all of the BoPGs. It is worth mentioning that the homologous protein of AtQRT3 (Bol028325), which was the only member of clade G, only had the II domain. In contrast, the III domain had the largest number of changes. All of the 21 members in clade E lacked the III domain. Among them, 14 (Bol037400, Bol041965, Bol370382, Bol045658, Bol042100, Bol014923, Bol014926, Bol013580, Bol033817, Bol036358, Bol009607, Bol042131, Bol013164, and Bol010852) only contained the I, II, and IV conserved domains; four (Bol016592, Bol033059, Bol034031, and Bol035965) only contained the I and II conserved domains; and two (Bol036900 and Bol022969) only contained the II conserved domain.

The gene structure was analyzed according to the DNA sequence and the cDNA sequence of the BoPGs (Figure 2b). It was found that the structure of exons–introns was highly variable among different clades of PG members. Clade C and clade D were relatively conserved. Clade C contained 18 members, 16 of which had five exons. Clade D had 24 members, 14 of which contained four exons. Compared with clade C and clade D, the exon–intron structures of the members in clade A, clade E, and clade F displayed large variations. Among them, the 21 members of clade E had the largest variation, and the number of exons varied from 1 to 6. In addition, according to the information on exons, we analyzed the phase of introns. The results showed that the phases were basically the same among the genes with a conservative exon–intron structure in each clade. For example, the intron phases of the 15 members in clade C, which had five exons, were 0, 2, and 0. The phases of introns of the 14 members with four exons in clade D were 0, 0, and 0. However, similar to the changes in gene structure, the intron phases among clade E PGs varied greatly.

**Figure 3:**
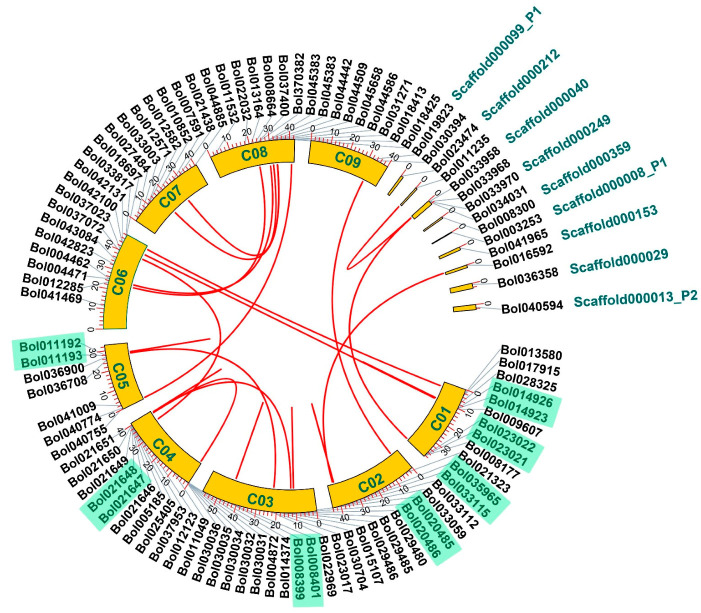
Genomic distribution and syntenic analysis of PG genes in *B. oleracea*. The chromosome numbers C01 to C09, and nine scaffolds are demonstrated by green letters. Tandem-duplicated genes are indicated by the green background. The red lines represent syntenic relationships of the paralogous BoPGs.

We analyzed the distribution and transcription direction of the BoPGs on the chromosomes. In total, 87 genes were localized on nine chromosomes (C01–C09), whereas the other twelve were on nine scaffold fragments (Figure 3 and Supplementary Materials Table S2). The numbers of the 87 members on chromosomes C01 to C09 were 9, 5, 12, 17, 7, 12, 8, 15, and 2. Among these, the transcription direction of 48 members on the chromosome was 5′~3′, and the remaining 39 genes were 3′~5′ (Supplementary Materials Table S2).

### 2.2. Expansion Mechanisms Accounting for the PG Family in B. oleracea

According to the results, we found that the distribution of PG genes on *B. oleracea* chromosomes appears to be nonrandom. According to the phylogenetic relationships of BoPGs (Figure 2), the clusters with high densities were observed on several chromosomes, especially chromosomes C03, C04, and C08. Moreover, seven sets of tandem replication gene pairs were located on five chromosomes (Figure 3). Among them, there were two sets on chromosome C01 (*Bol014926* and *Bol014923*; and *Bol023022* and *Bol023021*), one set on chromosome C02 (*Bol033115* and *Bol033112*), two sets on chromosome C03 (*Bol020485* and *Bol020486*; and *Bol008399* and *Bol008401*), one set on chromosome C04 (*Bol021647* and *Bol021648*), and one set on chromosome C05 (*Bol011192* and *Bol011193*). Among these replication gene pairs, there were four sets belonging to clade D (*Bol023022* and *Bol023021*; *Bol033115* and *Bol033112*; *Bol020485* and *Bol020486*; and *Bol008399* and *Bol008401*). The remaining three belonged to clade E (*Bol014926* and *Bol014923*), clade C (*Bol021647* and *Bol021648*), and clade F (*Bol011192* and *Bol011193*).

After splitting with *A. thaliana*, Brassicaceae species underwent an addition of whole genome triplication (WGT) [28]. Therefore, in theory, the genome size of *B. oleracea* should be three times the size of the *A. thaliana* genome. However, the WGT of Brassicaceae species in the evolution process involved substantial genomic rearrangement and gene loss, resulting in a large variation in the number of chromosomes and the size of the genome. The triplicated blocks resulting from WGT were partitioned into three subgenomes: the least fractionated subgenome (LF), medium fractionated subgenome (MF1), and most fractionated subgenome (MF2) [28,29,30]. In our study, more PGs were detected in *B. oleracea* than in *A. thaliana*, but far less than three times the number of *A. thaliana* PGs. By analyzing the syntenic PG genes between the two species and the distribution of BoPGs in three subgenomes, we found that 55 out of 68 PGs (80.9%) of *A. thaliana* were syntenic with BoPGs, and 76 out of 99 BoPGs (76.8%) can map to the three subgenomes (Supplementary Materials Table S4). The numbers of BoPGs on the three subgenomes (LF, MF1, and MF2) were 33, 23, and 20, respectively. In order to analyze the variation of BoPGs during evolution, we calculated the proportions of them retained in each of the three subgenomes relative to *A. thaliana*. We found that the LF subgenome retained 16.2% of the PGs found in *A. thaliana*, whereas the MF1 and MF2 subgenomes retained substantially lower proportions of retained genes (11.3% and 9.8%, respectively). These results suggested that many BoPGs were lost during the evolution of *B. oleracea*.

**Figure 4:**
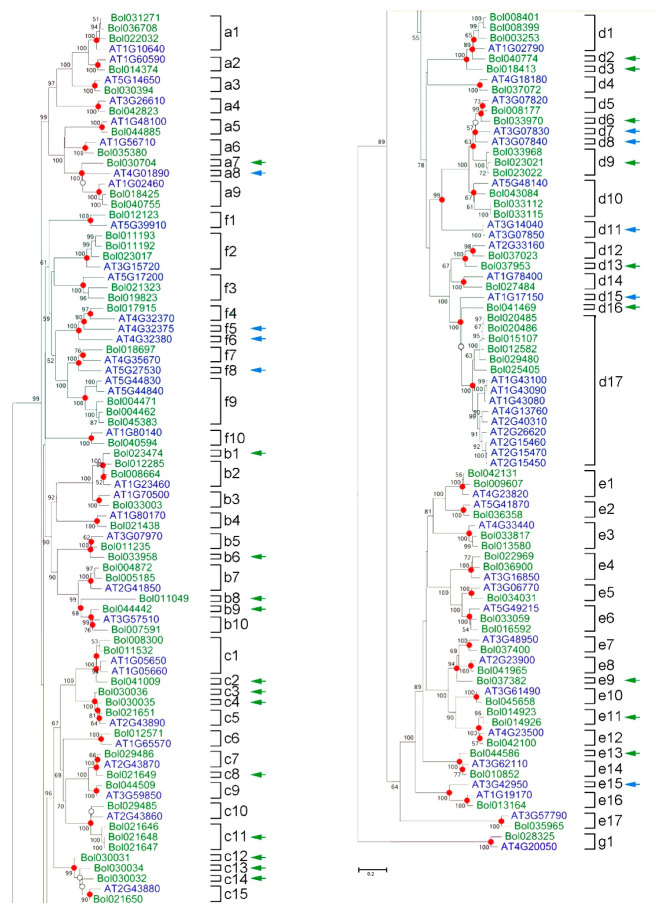
Phylogenetic tree of *B. oleracea* and *Arabidopsis thaliana* PGs. Numbers on branches indicate the bootstrap percentage values calculated from 1000 replicates, and only values higher than 50% are shown. The nodes that represent the most recent common ancestral genes before the *B. oleracea* and *A. thaliana* split are indicated by red dots (bootstrap support >50%) and white dots (bootstrap support <50%). Clades that contain only *B. oleracea* and *A. thaliana* PG genes are indicated by green and blue arrows, respectively.

Meanwhile, we analyzed the extent of lineage-specific expansion in *B. oleracea* and *A. thaliana*. According to the phylogenetic relationships, we identified the nodes that lead to *A. thaliana*–specific and *B. oleracea*–specific branches, which represented the most recent common ancestral genes before the split. During evolution, some PGs suffered from gene loss or gene gain. We found that 22 branches contained only BoPGs (Figure 4, green arrows), and nine branches contained only *Arabidopsis* PG genes (Figure 4, blue arrows), indicating that gene losses could have occurred in these branches. An analysis of the number of nodes on the phylogenetic tree, with 73 branches of common ancestral gene nodes containing higher confidence values (>50%) (Figure 4, red dots), indicated that there were at least 73 putative common ancestral genes before the isolation of *B. oleracea* and *A. thaliana*. In addition, there were six branches of common ancestor gene nodes with lower confidence values (<50%) (Figure 4, white dots). If these low confidence nodes are also correct, then *B. oleracea* and *A. thaliana* contain at least 79 putative common ancestral genes.

Based on phylogenetic analysis, the common ancestral genes of *B. oleracea* and *A. thaliana* obtained or lost during evolution were calculated, respectively (Supplementary Materials Figure S1). There were at least nine most recently common ancestral clade A PG genes in *B. oleracea* and *A. thaliana*. After the split, *B. oleracea* gained three genes and lost no genes. In contrast, *A. thaliana* gained no genes and lost one gene. Similarly, the numbers of genes gained and lost from the most recently common ancestral clade B to clade G PG genes in the *B. oleracea* lineage and *A. thaliana* lineage were calculated. The events of gene gain and loss have resulted in the rapid expansion of PG genes in these two plants. After the split, the number of genes gained in the *B. oleracea* lineage was greater than that in the *A. thaliana* lineage, except for clade G PGs, for which neither gain nor loss was observed in either species.

### 2.3. Expression Divergence Implies the Involvement of BoPGs in Different Tissues

**Figure 5:**
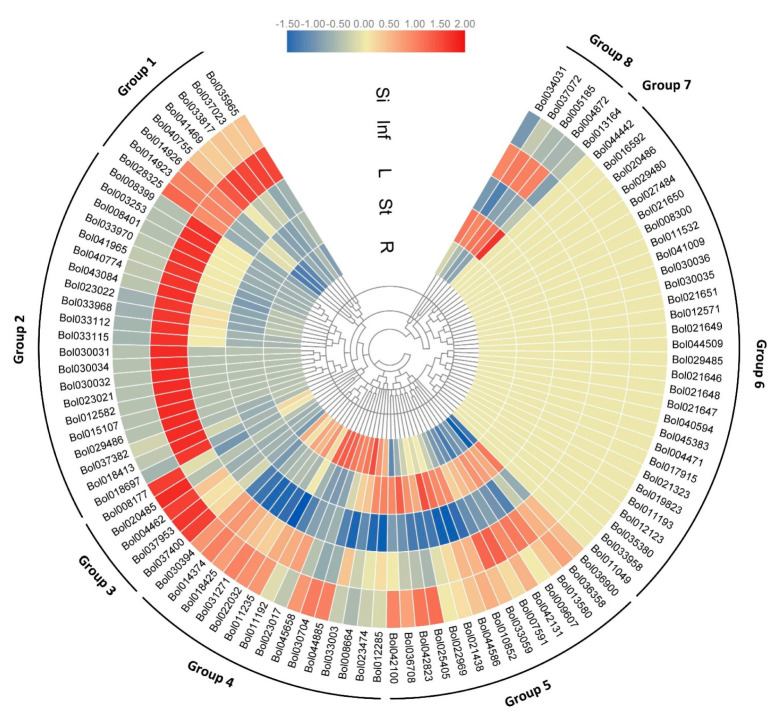
Hierarchical clustering and heatmap showing the expression levels of BoPGs. The scale bars represent the relative expression level. The blue, yellow, and red shading indicates a relatively low, medium, and high expression, respectively. Their expression characteristics are divided into eight groups. R: roots; St: stems; L: leaves; Inf: inflorescences; and Si: tender siliques.

We investigated the expression patterns of BoPGs by qRT-PCR under normal growth conditions. Firstly, we examined the expression of all 99 BoPGs in five tissues, including roots, stems, leaves, inflorescences, and tender siliques. We found that there was a substantially higher variation in the expression patterns between BoPGs. The result showed that all 99 BoPGs can be divided into eight groups (Group 1 to 8) according to their tissue specificity and relative gene expression levels (Figure 5). Group 1 contained eight genes, which had a higher expression level in inflorescences and tender siliques. Group 2 contained 21 genes whose expression could mainly be detected in inflorescences. Five members in Group 3 had a higher expression level in tender siliques. The expression of BoPG members in Group 4 and Group 5 displayed higher variation in roots, stems, inflorescences, and tender siliques, while almost all of them were not expressed in leaves. BoPG members in Group 6 did not express or had a very low expression level in every tissue. Group 7 contained one member, which was mainly expressed in one tissue—stems. The remaining four members that belonged to Group 8 were only expressed in inflorescences and stems. Taken together, approximately 30% of BoPGs did not express in any of the five tissue types tested, and almost all of the BoPGs did not express in leaves. Approximately 40% of expressed BoPGs had a high level of expression in inflorescences, 5% in root tissues, 8% in stems, and 14% in tender siliques. These findings indicate that most of the expressed BoPGs have a higher expression level in reproductive tissues (inflorescences and tender silique).

The expression patterns of BoPGs were also analyzed according to the seven distinct clades (clades A–G) (Supplementary Materials Figure S2). According to our result, the expressed BoPGs in clades C, D, and F showed a higher expression level in reproductive tissues (inflorescences and tender silique), while the expressed BoPGs in clades A, B, and E showed ubiquitous expression patterns. In particular, the 21 members in clade E exhibited all the expression characteristics of the eight groups. The only one member of clade G was specifically expressed in stems.

According to the results presented above, many BoPGs had higher expression levels in inflorescences tissue. To further elucidate their expression patterns in reproductive growth, we selected seven BoPGs which were specifically and largely expressed in inflorescences to examine their expression level in the flower buds (Bud 1 to Bud 5) (Supplementary Materials Figure S3). The result indicated that five members (*Bol370382*, *Bol041965*, *Bol033968*, *Bol023022*, and *Bol018697*) had the highest expression level in Bud 5; one member (*Bol035965*) had higher expression levels in Bud 1, Bud 2, and Bud 3; one member (*Bol033970*) showed a high expression level in Bud 1 and Bud 2; and one member (*Bol040774*) was only expressed in Bud 3. Subsequently, the five members, which were specific and dominantly expressed in Bud 5, were selected to further analyze their expression patterns in sepal, petal, filament, anther, and pistil of Bud 5. Among them, two members (*Bol041965* and *Bol018697*) were specific and dominantly expressed in the anther (Supplementary Materials Figure S4).

The expansion of the PG family raises an intriguing question on the mechanisms of duplicate retention and their functions in plants [31]. There may be similar expression patterns between more closely related genes. To test this, we investigated the expression patterns of 21 paralogous gene pairs which were at the terminal nodes of the phylogenetic tree, and the similarity was higher than 80% for identification analysis. By comparing the temporal and spatial expression characteristics, it was found that the expression differences of them were classified into four categories (I, II, III, and IV) (Figure 2 and Table 1). In the I category, the gene pairs displayed the same expression characteristic, which did not express (*Bol033112* and *Bol033115*; *Bol008399* and *Bol008401*; and *Bol010852* and *Bol044586*) or expressed in the same organ and with the same expression level (*Bol021648* and *Bol021647*; and *Bol021323* and *Bol019823*). In the II category, the gene pairs were expressed in the same organ but with a divergent expression level. Seven pairs belonged to this category. In the III category, the gene pairs were selectively expressed in different tissue or exhibited partial overlap. One pair displayed this expression pattern. In the IV category, one gene was not expressed, and the other was selectively expressed in certain tissue. Eight gene pairs belonged to this category. Taken together, among the 21 paralogous gene pairs, 16 pairs (76.2%) showed divergent expression patterns. These findings suggest that most of the retained gene pairs exhibited expression divergence.

To explore the relationship between the nonsynonymous substitution rates (*Ka*), synonymous substitution rates (*Ks*), and expression pattern, *Ka*, *Ks*, and *Ka*/*Ks* of the 21 paralogous gene pairs were calculated (Table 1). The result showed that the *Ka*/*Ks* values of 20 pairs were less than 1, indicating that these genes suffered negative selection during evolution. There was one pair (*Bol033115* and *Bol033112*) with *Ka* and *Ks* values equal to 0, indicating that the base has not been substituted during evolution. However, according to our result, there was not a significant correlation between the *Ka*/*Ks* and the gene expression divergence.

### 2.4. Cis-Elements Analysis of the BoPG Promoters

**Figure 6:**
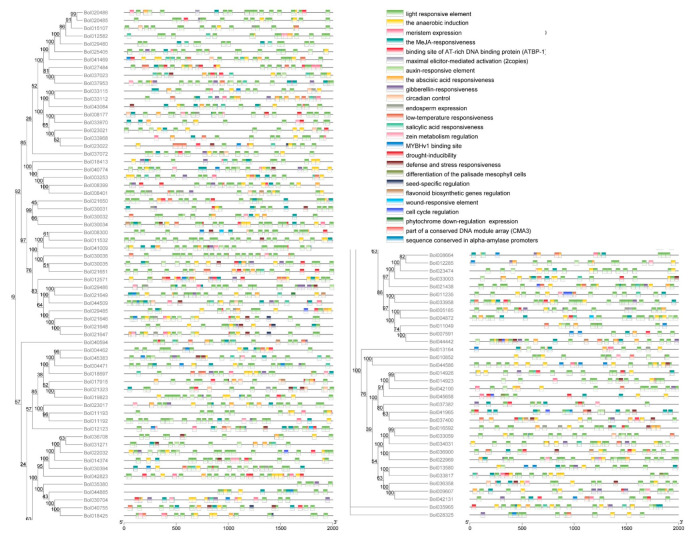
The cis-acting elements predication in the 2 kb sequences upstream of the “ATG” of the BoPGs. The types of elements are marked by different background colors.

Promoters can control the gene expression at the right time, place, and level [32]. To further investigate the potential functions of BoPGs, the cis-elements in their promoter sequences were identified and analyzed. Twenty-seven cis-elements related to biotic and abiotic stresses, as well as hormone responses, were detected (Figure 6 and Supplementary Materials Tables S5 and S6). Up to 96 promoters contained the hormone-response cis-elements (the MeJA-responsiveness (CGTCA-motif, and CGTCA-motif), auxin-responsive element (TGA-box and AuxRR-core), gibberellin-responsiveness (GARE-motif, P-box, and TATC-box), salicylic acid responsiveness (TCA-element), or abscisic acid responsiveness (ABRE)). In terms of the responsiveness to various abiotic stresses, 45 promoters containing the cis-acting element related to low-temperature responsiveness (LTR) and 36 promoters, including the drought-inducibility element (MBS) were identified. All of the promoters of BoPGs contained a light-responsive element. The MYBHv1 binding sites (CCAAT-box) were detected in 33 promoters. The cis-element essential for anaerobic induction (anaerobic responsive element, ARE) was detected in up to 86 promoters, whereas only three promoters covered the wound-responsive element (wound (WUN)-motif). In addition, 37 promoters containing zein metabolism regulation elements (O2-site) were identified. Flavonoid biosynthetic gene regulation elements were detected in 10 promoters. There were seed-specific regulation elements (RY-element) and root-specific elements (motif I) in six and one promoters, respectively. In general, 27 kinds of cis-elements were detected in the promoters of 99 BoPGs.

### 2.5. Subcellular Localization of BoPG Proteins

**Figure 7:**
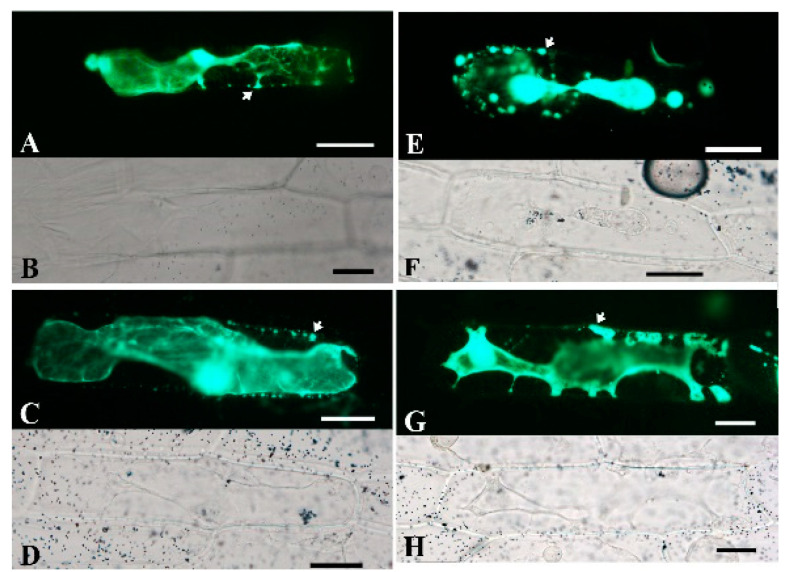
Subcellular localization of four BoPG-green fluorescent protein (GFP) fusion proteins in onion epidermal cells. (**A**, **E**, **C**, and **G**) show fluorescence images of plasmolyzed transgenic cells. The fluorescence signal can be observed in the cell wall (white arrows). (**B**, **F**, **D**, and **H**) are bright-field images of the corresponding onion epidermal cells. Scale bars = 50 μm.

The PGs localize to the plant cell wall and are involved in the degradation of pectin [33]. The presence of the amino acid N-terminal precursor sequence keeps the PG protein in an inhibitory state until it is transported to the final site of action, or localizes the PG protein to a specific site on the cell wall [34]. In this study, TargetP 1.1 Server was used to predict the subcellular location of eukaryotic proteins (Supplementary Materials Table S2). All of the 99 BoPG proteins were predicted to contain a signal peptide, indicating that these proteins might be secreted proteins. To examine the accuracy of the prediction of subcellular locations of BoPGs, we selected the four PG proteins (Bol041965, Bol035965, Bol040774, and Bol041469), which had a higher expression level in inflorescences to construct subcellular localization fusion expression vectors. After transient expression of the fusions in onion epidermis cells, we visualized the green fluorescent protein (GFP) signal by confocal microscopy. Observations indicated that the fluorescent signals of the four proteins were ubiquitously distributed in the onion epidermal cells. To determine if these proteins could be localized on the cell wall, we performed a plasmolysis experiment by treatment with 0.3 g·mL^−1^ sucrose. After plasmolysis, the fluorescent signals could be observed in the cell wall (Figure 7). The results above indicated that the four PG proteins were secreted proteins and could localize on the cell wall.

## 3. Discussion

PGs play a key role in degrading pectin of the cell wall in plant development. The gene family of PG has recently been identified in many higher plants. Therefore, it is understandable that PG is a very important enzyme in the study of cell wall hydrolysis. Here, we identified 99 full-length PG genes from *B. oleracea*. Phylogenetic trees were developed to study the evolution of PGs between *B. oleracea* and *A. thaliana*. Furthermore, based on qRT-PCR data, BoPGs displayed specific temporal and spatial expression patterns, which provided clues for investigating their specific functions.

The phylogenetic analysis constructed by amino acid sequences from five spices showed that all of the BoPGs were clustered into seven clades. This is similar to the results in other species, such as *B. rapa* [21] and *S. lycopersicum* [8]. By analyzing the PG numbers of the five species in different clades, we can speculate that clades A, B, and E contained PGs of land plants, and clade C and clade D only included PG genes of flowering plants. This result was consistent with the report of Park et al. [18]. According to our result, clade G only contained the PGs of dicotyledons. This may indicate that PG genes in clade G appeared later during evolution, while clades A, B, E, and F are more primitive. Meanwhile, in our result, the rapid expansion events of PG genes were found from monocotyledons to dicotyledons, which might have resulted from lineage-specific expansion after the split of monocotyledonous plants from their dicotyledonous counterparts about 140–150 Mya [35]. Furthermore, the PG expansion occurred after the split of lycophytes to euphyllophytes.

Divergences in coding regions, especially those that can change the function of the gene, can be caused by amino acid altering substitutions and/or alterations in the exon–intron structure [27]. In our study, the exon numbers of the 21 members in clade E exhibited the largest variation, varying from 1 to 6. When we analyzed the expression characteristics of BoPGs in different clades, we found that the expressed BoPGs of clade E showed ubiquitous expression characteristics. Therefore, the changes of the exon–intron structure might relate to the expression of BoPGs. It is possible that PG functional divergence can be, in part, attributed to expression divergence [36]. Therefore, it can be speculated that the BoPGs in clade E might participate in multiple stages of the growth and development of *B. oleracea*. This further proved the previous theory that the clade E members of PG family are possibly ancient proteins and are fundamental and indispensable in almost all plant organs of different species [18]. It is worth mentioning that clade F members have been shown to maybe be related to flowering and probably fruit development in previous studies [18,21]. In our study, the expressed BoPGs in clades D, C, and F were mainly responsible for reproduction development (inflorescences or tender siliques). Moreover, the BoPG *Bol018697* that belonged to clade F was dominantly expressed in Bud 5 in our result. This BoPG has been demonstrated to be closely related to pollen development by in situ hybridization and promoter expression analysis [14]. The possible functions of other BoPGs, especially the genes with higher expression levels, need further study.

According to previous research reports, domains I and II may be components of the catalytic site, domain III is thought to participate in the catalytic reaction, and domain IV (RIK) constitutes a possible candidate for interactions with the ionic groups of the carboxylic acid groups in the substrate [16,37,38,39]. In our results, almost all of the BoPGs in clades A, B, C, D, and F contained all of the four conserved domains, while all of the clade E members lacked the III domain. This is similar to the results obtained for other species, with domain III showing lower conservation and being missing in clade E PGs [8,20,21]. The reason why the expression characteristics of clade E PGs displayed large variation may be associated with the lack of the III domain. In addition, some BoPG members only have the two (I and II) or one (II) domain. This indicates that these genes might have catalytic activity, but lost their interaction ability, with the substrate containing the ionic groups of the carboxylic acid groups. Since the protein domain is closely related to its function, the II domain may be more closely related to the identification of PG.

After splitting with *A. thaliana*, Brassicaceae underwent the addition of WGT, which was thought to have occurred between 13 and 17 million years ago [28,29]. The WGT of Brassicaceae species in evolution involved genomic rearrangement and gene loss [28,40]. The number of BoPGs was 99, which was far less than three times the number of *A. thaliana* PGs. Moreover, by analyzing the proportions of BoPGs retained in each of the three subgenomes relative to *A. thaliana*, we found that most of the BoPGs were lost during evolution. Therefore, we speculated that gene loss may be an important mechanism in the generation of diversity of BoPGs. This was consistent with the result obtained from *Populus*, showing that gene loss might be a general evolutionary pattern of large gene family evolution [19,41]. Furthermore, the seven tandem-duplicated pairs were only present in C01–C05, indicating that the expansion modes of BoPGs in different chromosomes after splitting with *A. thaliana* were not the same during the evolutionary process. After calculating the obtained or lost BoPGs in different clades when split with *A. thaliana*, we found that their numbers in clade A to clade E were all changed. In particular, the tandem repeat event mainly occurred in clade C and clade D. This illustrated that there are also differences in the expansion mode of BoPGs in different clades.

By comparing the common ancestor genes of different clades and the number of BoPGs obtained or lost during evolution, we found that, although a large number of PG genes were lost, many duplicates have been retained in the *B. oleracea* genome. The retention and loss of duplicate genes are related to the functional needs of species. In general, the variation in expression is believed to be the initial step in functional divergence among duplicate genes and thereby increases the probability of the existence of duplicate genes in the genome [42]. Moreover, the phenomena of neofunctionalization and subfunctionalization may also have been adapted by the retained duplicate genes. The neofunctionalization model postulates that the gain of new function(s) is a major factor in the retention of both copies of duplicate genes in a genome. The subfunctionalization model is also known as the duplication-degeneration-complementation (DDC) model, and it assumes that the two duplicate genes undergo complementary degeneration of their cis-regulatory motifs, so that both copies are required to produce the full complement of the cis-regulatory motifs of the ancestral gene [36,42,43]. By analyzing the temporal and spatial expression characteristics of 21 paralogous BoPG gene pairs, 16 pairs (76.2%) showed divergent expression patterns. According to our statistics, some paralogs were selectively expressed in different tissues or partial overlap, indicating that they may have undergone neofunctionalization in the process of evolution, or that both of them have undergone subfunctionalization and have complementary functions for the growth and development of plants. In addition, in some paralogs, one gene was not expressed, and the other was selectively expressed in certain tissue. This might be due to one of the paralog genes having become a pseudogene or the other one having obtained a new function during the process of evolution. Moreover, some paralogs displayed the same expression characteristic (did not express or expressed in the same organ and with the same expression level), indicating that the two genes may both be pseudogenes or have the same function. 

Transcriptional regulation is a crucial contributor to evolutionary change in the genotype–phenotype relationship [32]. At the transcription level, the combination of cis-acting elements and transcription factors in the promoter region is involved in the regulation of gene expression. For example, two duplicated PG genes, *BcMF26a* and *BcMF26b*, in *B. rapa*, with amino acid sequences, had 92.19% similarity and showed a divergent expression and function, which may be responsible for their promoters having a very large divergence (63.20% similarity) [44]. In our study, the cis-elements in BoPGs’ promoter sequences were identified and analyzed. We found that up to 96 promoters contained the hormone response cis-elements, including ABA, IAA, MeJA, and GA, and different stresses. Some papers have characterized the relationship between PG and hormones. Hou et al. [45] and Lin et al. [46] showed that the inhibitor of ethylene perception 1-methylcyclopropene (1-MCP) can distinctly suppress the expression levels of PaPG1 and decrease the PG activities of apricots and plums during cold storage. During the germination of tomato seeds, there is a delay in ABA degradation, and the activity of the four cell-wall-degrading enzymes (including exo-polygalacturonase) evaluated is inhibited [47]. Moreover, previous research results showed that ethylene, JA, and ABA were required for the normal expression of the PG gene *QRT2* in floral organ abscission zones. While ethylene may directly regulate *QRT2* expression in the floral organ abscission zones, JA and ABA may act indirectly via roles in promoting flower senescence [48]. Therefore, the hormones may participate in plant development by regulating the PGs’ activities. However, relevant research reports are still lacking, especially on reproductive development. According to our result, hormones may also be important for the growth and development of *B. oleracea*. However, the regulation of PGs’ function by hormone signaling needs to be further clarified.

## 4. Materials and Methods

### 4.1. Identification and Sequence Analysis

Gene identifiers of 68 *A. thaliana* PG genes were obtained from Liang et al. [21]. The corresponding protein sequences were obtained from The Arabidopsis Information Resource (http://www.arabidopsis.org/). The target PG family members were searched for using the *B. oleracea* genome database (http://www.ocri-genomics.org/bolbase/index.html.) in the TBLASTP program, with default algorithm parameters. Then, together with the flank regions of 2 kb upstream and downstream, the candidates were re-annotated by using FGENESH (http://linux1.softberry.com/berry.phtml?group=programs&subgroup=gfind&topic=fgenesh). All *B. oleracea* PG candidates collected were primarily analyzed by using the protein families’ database (Pfam) to confirm the presence of GH28 domains in their protein structures. Candidate genes with at least one PG conserved domain (domain I, II, III, and IV) were collected. The well-known PG AtQRT3 [49], which does not contain one conserved domain of PG, was also analyzed.

The whole sequences of chromosomes and scaffolds were downloaded from the *B. oleracea* database. Together with the results of BLASTP, we obtained information of genomic sequences, cDNA sequences, intron distribution patterns, phases, and intron/exon boundaries of the deduced BoPGs. Together with the sequence information provided above, we located all of the BoPGs on the chromosomes and scaffolds. An exon/intron map was constructed by using the software TBtools. The conserved domains were analyzed, using the MEME website (http://meme.sdsc.edu/meme/meme.html). Their protein molecular weight and isoelectric point were predicted by using PROTEIN CALCULATOR v3.4 (http://protcalc.sourceforge.net/). Their signal peptide sequence prediction was performed by using SignalP-5.0 Server (http://www.cbs.dtu.dk/services/SignalP/). 

To analyze the cis-acting elements, we first obtained the *B. oleracea* genome sequence from the Bolbase (http://www.ocri-genomics.org/bolbase/index.html). By using the TBtools software, we extracted and obtained 2 kb sequences upstream of the BoPGs’ start codon. Then, the cis-elements in the promoter sequences were predicted by PlantCare (http://bioinformatics.psb.ugent.be/webtools/plantcare/html/). The predicted cis-acting elements information was visualized by using TBtools software.

### 4.2. Phylogenetic and Molecular Evolution

Multiple sequence alignment for full-length PG protein sequences of five species was generated by using ClustalX. The full-length PG protein sequences of *P. patens* (moss and bryophyte), *S. moellendorffii* (spikemoss and lycophyte), and *O. sativa* were obtained from a previous study [6,19]. Phylogenetic relationships were developed by the MEGA7 program, using a neighbor-joining (NJ) procedure with the following parameters: Poisson correction, pairwise deletion, and bootstrap analysis with 1000 replicates. Sequence gaps were treated as missing characters. Then, the online software iTOL (https://itol.embl.de/) was used to decorate the phylogenetic tree. According to the method of Park et al. [18], the different clades were divided.

*Brassicaceae* species have three subgenomes which share the same diploid ancestor of the model species *A. thaliana*. Among these three subgenomes, there are biased gene fractionation (gene loss), named the least fractionized subgenome as LF, the moderate gene fractionized as MF1, and the most gene fractionized as MF2. According to the report of Cheng et al. [50], we searched for syntenic genes between *A. thaliana* and *B. oleracea*, using the “Syntenic gene search” in the Brassica Database (http://brassicadb.org/brad/searchSynteny.php). The distribution of syntenic BoPGs of *A. thaliana* in the three subgenomes was recorded.

The phylogenetic relationship between *B. oleracea* and *A. thaliana* was analyzed by the MEGA7 program by using a neighbor-joining (NJ) procedure with the following parameters: Poisson correction, pairwise deletion, and bootstrap analysis with 1000 replicates. Using the method of Kim et al. [31], the extent of lineage-specific expansion of the PG genes between the two species was investigated. The branches were defined by identifying nodes representing speciation events (dots). For those branches, a bootstrap value higher than 50% indicated the criteria for the least number of common ancestral PGs between *B. oleracea* and *A. thaliana*. The branches containing only sequences for one of the two plants indicated that gene loss had occurred during evolution. 

In this study, when the length of highly similar sequences between two closely linked genes was more than 80% of the longer one, whilst their similarity was greater than 80%, they were characterized as tandem replication genes. The nonsynonymous substitution rates (*Ka*) and synonymous substitution rates (*Ks*) between paralog pairs within BoPGs were calculated by using the *KaKs* Calculator program of TBtools software. Additionally, by using TBtools, we conducted synteny analysis and generated a diagram of the genomic distribution in *B. oleracea.*

### 4.3. Gene Expression Analysis

*B. oleracea* cv. Sanxiong plants were cultivated on the experimental farm of the Zhejiang Academy of Agricultural Sciences. Flower buds (Bud 1 to Bud 5) at five pollen developmental stages (stage 1, pollen mother cell stage; stage 2, tetrad stage; stage 3, uninucleate microspore stage; stage 4, binucleate microspore stage; and stage 5, mature pollen stage) were selected by cytological detection, as described by Huang et al. [51]. At the flowering stage, the samples, including flower buds (Bud 1 to Bud 5), five different organs (roots, stems, leaves, inflorescences, and germinal siliques), and five floral parts (sepals, petals, filaments, anthers, and pistils) of Bud 5 were collected and stored at −80 °C.

The total RNA of the above samples was purified by using TRIzol reagent (Invitrogen, Carlsbad, CA, USA). First-strand cDNA was synthesized by using the PrimeScript RT Reagent Kit (TaKaRa, Dalian, China), in accordance with the manufacturer’s instructions.

The qRT-PCR analysis was used to examine the transcript levels of BoPGs. Based on the multiple-sequence alignment of BoPG gene sequences, specific primers were designed by using Primer premier 5.0 (Supplementary Materials Table S7). The primers of the PG genes which have a high sequence similarity were designed in the non-conserved regions of the gene coding sequence or the 5′/3′-untranslated region for expression analysis. Two highly similar genes that do not have large-scale different sequences suitable for primer design, respectively, were used for the same primer pairs for the qRT-PCR (the five sets with a yellow back label in Supplementary Materials Table S7). The constitutively expressed gene *GAPDH* [52] was selected as an internal control. The qRT-PCR was performed with the CFX96 Real Time System (Bio-Rad, California, Hercules, USA) machine. PCR conditions were 30 s at 95 °C, followed by 40 cycles of 5 s at 95 °C, 30 s at 65 °C, and 1 min at 72 °C. Each sample was run in triplicate with 15 μL of the reaction volume by using the SYBR Premix Ex Taq Kit (TaKaRa, Dalian, China). For qRT-PCR analysis, three technical replications were applied. Expression analysis of low- and non-expressed genes was conducted at least twice with independent experiments. The 2^-ΔΔCt^ method was used to calculate the relative expression levels. The software TBtools was employed to generate the heatmap.

### 4.4. Subcellular Localization

We selected four BoPGs which had higher expression levels in inflorescences, to investigate their subcellular localizations by generating C-terminal green fluorescent protein (GFP) fusions. Based on their CDS provided by the *Brassica* database, we designed four specific primers (Supplementary Materials Table S8) for nucleotide fragment amplification. The PCR amplification reaction was carried out by using the high-fidelity enzyme KOD-plus-Neo, in accordance with the manufacturer’s instruction. The resulting fragment was cloned into the fusion expression vector pFGC–GFP under the control of the constitutive CaMV35S promoter, to create the fusion vector. Colonies containing the appropriate insert were identified by sequencing. The fusion vector was then transiently transformed into onion epidermal cells by particle bombardment. The fluorescence signal was analyzed after 16 h of incubation with the Nikon Eclipse 90i Fluorescent Microscope (ECLIPSE 90i; Nikon). The onion epidermal cells were plasmolyzed by infiltrating 0.3 g·mL^−1^ sucrose.

## Figures and Tables

**Table 1:** The synonymous substitution rate (Ka) and nonsynonymous substitution rate (Ks) between paralogous PG gene pairs of *B. oleracea*. Gene expression patterns were categorized into four categories (I, II, III, and IV).

Paralogous Gene Pairs		*Ka*	*Ks*	*Ka*/*Ks*	Categories
*Bol020486*	*Bol020485*	0.0021	0.0264	0.0808	IV
*Bol037023*	*Bol037953*	0.1202	0.5223	0.2301	II
*Bol033115*	*Bol033112*	0	0	——	I
*Bol008177*	*Bol033970*	0.0547	0.267	0.2047	II
*Bol008399*	*Bol008401*	0.0221	0.0661	0.3347	I
*Bol029486*	*Bol021649*	0.0383	0.2782	0.1377	IV
*Bol021648*	*Bol021647*	0.0063	0.0072	0.8854	I
*Bol004462*	*Bol045383*	0.0316	0.1065	0.297	IV
*Bol021323*	*Bol019823*	0.1487	0.3771	0.3942	I
*Bol011193*	*Bol011192*	0.0504	0.1386	0.3636	IV
*Bol040755*	*Bol018425*	0.0359	0.2634	0.1361	II
*Bol008664*	*Bol012285*	0.0383	0.367	0.1044	II
*Bol011235*	*Bol033958*	0.0934	0.3071	0.3041	IV
*Bol005185*	*Bol004872*	0.0471	0.2669	0.1766	I
*Bol007591*	*Bol044442*	0.1061	0.323	0.3283	IV
*Bol010852*	*Bol044586*	0.0528	0.3528	0.1498	I
*Bol014926*	*Bol014923*	0.003	0.0152	0.201	IV
*Bol016592*	*Bol033059*	0.0526	0.3394	0.155	IV
*Bol036900*	*Bol022969*	0.0622	0.2988	0.2082	II
*Bol013580*	*Bol033817*	0.0427	0.3785	0.1129	III
*Bol009607*	*Bol042131*	0.0294	0.3163	0.0928	II

## Supplementary Materials

Supplementary Materials can be found at https://www.mdpi.com/1422-0067/21/16/5706/s1. Figure S1. The copy number changes of *B. oleracea* and *A. thaliana* PG genes in A to G clades. Numbers in ellipses and rectangles represent the numbers of PG genes in extant and ancestral species, respectively. Numbers on branches with plus and minus symbols represent the numbers of gene gains and losses, respectively. Figure S2. Hierarchical clustering and heat map showing the expression levels of BoPGs in seven clades. The scale bars represent relative expression level. The blue, yellow, and red shading indicate relatively low, medium, and high expression respectively. R: roots, St: stems, L: leaves, Inf: inflorescences, Si: siliques. Figure S3. The expression analysis of BoPGs which had specific or high expression levels in inflorescences. B1 (Bud1) - B5 (Bud5) indicate the flower buds at different pollen development stages. B1, pollen mother cell stage; B2, tetrad stage; B3, uninucleate microspore stage; B4, binucleate microspore stage; and B5, mature pollen stage. Figure S4. The expression analysis of *Bol041965* and *Bol018697* in the five organs of Bud5. Se, Sepals; Pe, petals; Fil, Filaments; An, Anthers; Pi, pistils. Table S1. Full-length amino acid sequence of PGs identified from B. oleracea genome. Table S2. The molecular details analysis of BoPGs. Table S3. The number of PG members of cladeA ~ clade G in five terrestrial plant species. Table S4. The syntenic PG genes between B. oleracea and A. thaliana and the distributed of BoPGs in three subgenomes (LF, MF1 and MF2). The orange background indicates the recurring PG genes. Table S5 & Table S6. The cis-element analysis of BoPGs’ promoters. Table S7. Primers used in qRT-PCR analysis of PG genes in B. oleracea. The sets with yellow background indicate the genes used the same primer pairs for qRT-PCR. Table S8. The four pairs of primer sequences for PCR amplification in subcellular localization experiment. The underlined base represents the cleavage site.
