# Supplementary material for: Patterns of Expansion and Expression Divergence of the Polygalacturonase Gene Family in Brassica oleracea
Source: Int J Mol Sci. 2020 Aug 9;21(16):5706. doi: 10.3390/ijms21165706 (PMC7461206; doi:10.3390/ijms21165706)
Supplement: Supplementary file 1 [file ijms-21-05706-s001.zip › ijms-851976 supplementary check/Figure S1-S4.pdf]

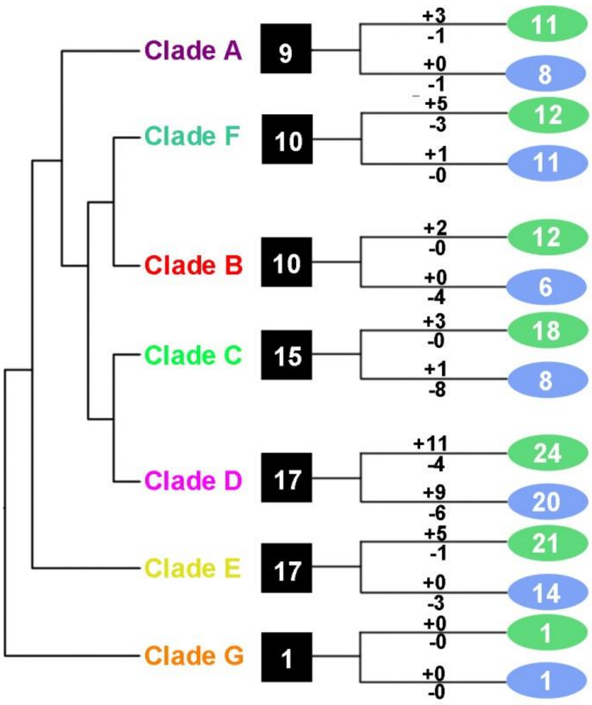

Figure S1. The copy number changes of *B. oleracea* and *A. thaliana* PG genes in A to G clades. Numbers in ellipses and rectangles represent the numbers of PG genes in extant and ancestral species, respectively. Numbers on branches with plus and minus symbols represent the numbers of gene gains and losses, respectively.

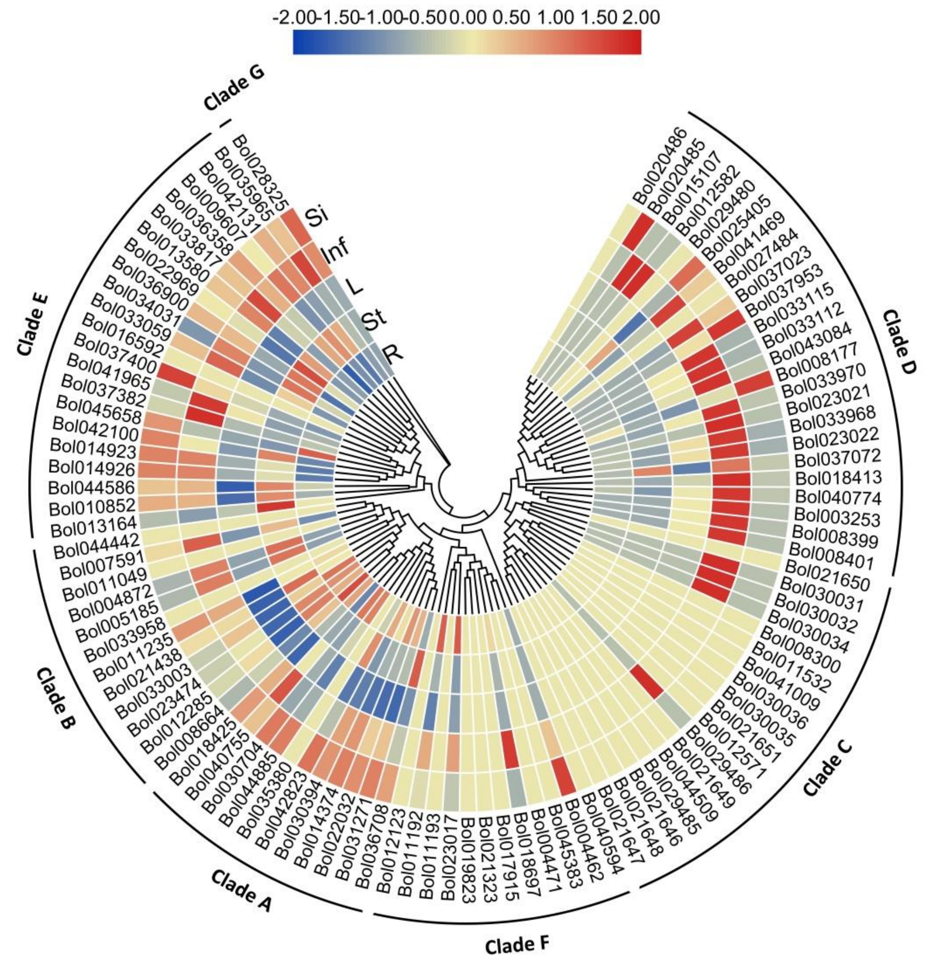

Figure S2. Hierarchical clustering and heat map showing the expression levels of BoPGs in seven clades. The scale bars represent relative expression level. The blue, yellow, and red shading indicate relatively low, medium, and high expression respectively. R: roots, St: stems, L: leaves, Inf: inflorescences, Si: siliques.

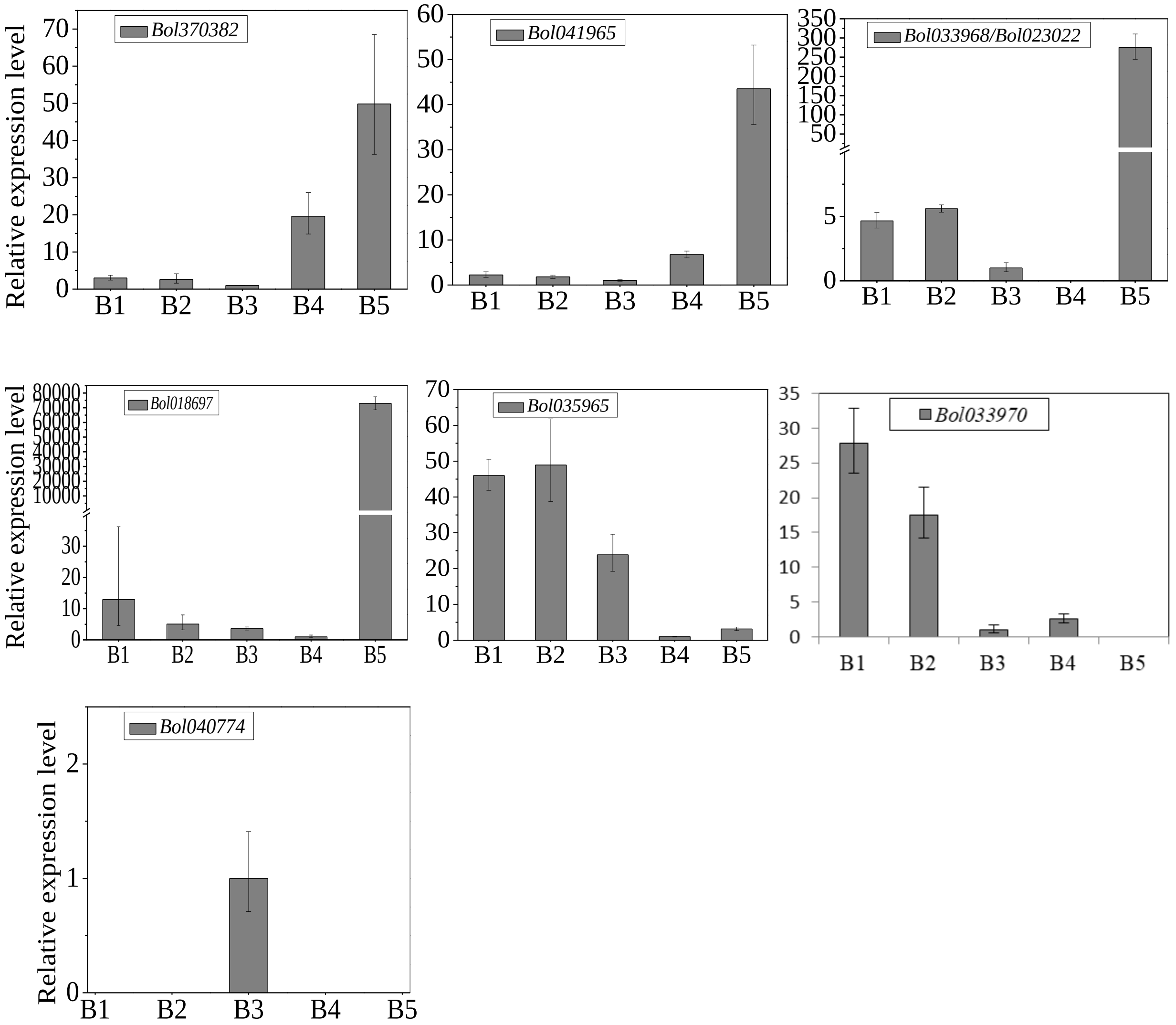

Figure S3. The expression analysis of BoPGs which had specific or high expression levels in inflorescences. B1 (Bud1) - B5 (Bud5) indicate the flower buds at different pollen development stages. B1, pollen mother cell stage; B2, tetrad stage; B3, uninucleate microspore stage; B4, binucleate microspore stage; and B5, mature pollen stage.

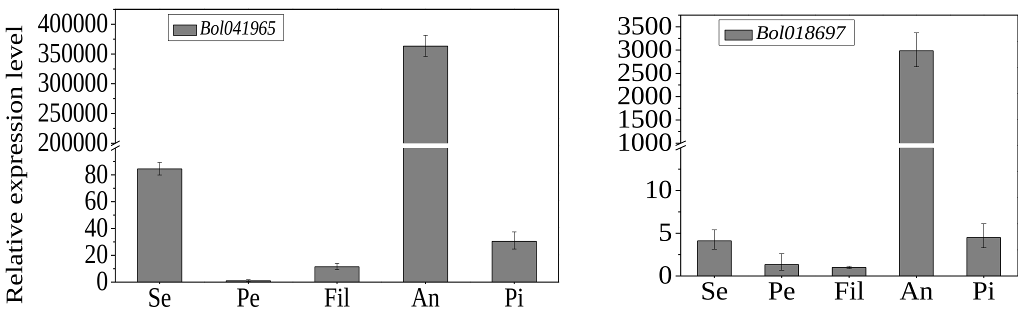

Figure S4. The expression analysis of *Bol041965* and *Bol018697* in the five organs of Bud5. Se, Sepals; Pe, petals; Fil, Filaments; An, Anthers; Pi, pistils.
